# Association of progesterone receptor status with 21-gene recurrence score and survival among patients with estrogen receptor-positive breast cancer

**DOI:** 10.1186/s12885-023-10796-4

**Published:** 2023-04-11

**Authors:** Sung Jun Ma, Jasmin Gill, Keerti Yendamuri, Udit Chatterjee, Olivia Waldman, Cynthia Dunne-Jaffe, Fatemeh Fekrmandi, Rohil Shekher, Austin Iovoli, Song Yao, Oluwadamilola T. Oladeru, Anurag K. Singh

**Affiliations:** 1 Department of Radiation Medicine, Roswell Park Comprehensive Cancer Center, 665 Elm Street, Buffalo, NY 14203 USA; 2 University at Buffalo, The State University of New York 12 Capen Hall, Buffalo, NY 14260 USA; 3 Jacobs School of Medicine and Biomedical Sciences, University at Buffalo, The State University of New York, 955 Main Street, Buffalo, NY 14203 USA; 4 Department of Cancer Prevention and Control, Roswell Park Comprehensive Cancer Center, 665 Elm Street, Buffalo, NY 14203 USA; 5 Department of Radiation Oncology, University of Florida, 2000 SW Archer Road, Gainesville, FL 32608 USA

**Keywords:** PR status, Oncotype score, Chemo, Chemotherapy

## Abstract

**Background:**

Progesterone receptor (PR)-negative tumors have been shown to have worse prognosis and were underrepresented in recent trials on patients with estrogen receptor (ER)-positive breast cancer. The role of PR-negative status in the context of 21-gene recurrence score (RS) and nodal staging remains unclear.

**Methods:**

The National Cancer Database (NCDB) was queried for women diagnosed between 2010 and 2017 with ER-positive, human epidermal growth factor receptor 2 (HER2)-negative, pT1-3N0-1a breast cancer. Logistic and Cox multivariable analyses (MVA) were performed to identify association of PR status with high RS (> 25) and overall survival (OS), respectively.

**Results:**

Among 143,828 women, 130,349 (90.6%) and 13,479 (9.4%) patients had PR-positive and PR-negative tumors, respectively. Logistic MVA showed that PR-negative status was associated with higher RS (> 25: aOR 16.15, 95% CI 15.23–17.13). Cox MVA showed that PR-negative status was associated with worse OS (adjusted hazards ratio [aHR] 1.20, 95% CI 1.10–1.31). There was an interaction with nodal staging and chemotherapy (*p* = 0.049). Subgroup analyses using Cox MVA showed the magnitude of the chemotherapy benefit was greater among those with pN1a, PR-negative tumors than pN1a, PR-positive tumors (PR-positive: aHR 0.57, 95% CI 0.47–0.67; PR-negative: aHR 0.31, 95% CI 0.20–0.47). It was comparable among those with pN0 tumors regardless of PR status (PR-positive: aHR 0.74, 95% CI 0.66–0.82; PR-negative: aHR 0.63, 95% CI 0.51–0.77).

**Conclusion:**

PR-negative tumors were independently correlated with higher RS and were associated with greater OS benefits from chemotherapy for pN1a tumors, but not pN0 tumors.

**Supplementary Information:**

The online version contains supplementary material available at 10.1186/s12885-023-10796-4.

## Introduction

Among patients with estrogen receptor (ER)-positive, human epidermal growth factor 2 (HER2)-negative breast cancer, progesterone receptor (PR)-negative tumors were shown to have worse prognosis than PR-positive tumors [1–3]. PR-negative tumors have been shown to be less likely to respond to endocrine therapy than PR-positive tumors [1, 4, 5], and treatment intensification with adjuvant chemotherapy may improve the outcome of PR-negative tumors [6].

However, PR-negative tumors were underrepresented in recent trials that led to a routine use of 21-gene recurrence score (RS) in clinical practice, and they were only 10.0% and 5.7% of all tumors included in TAILORx and RxPONDER trials, respectively [7, 8]. Findings from these trials may not be generalizable to PR-negative tumors. In addition, a recent guideline by the American Society of Clinical Oncology (ASCO)/College of American Pathologists (CAP) recognized the heterogeneity in the extent of staining for ER and PR status, encouraging them to be further stratified by negative, low positive, and positive based on 1% and 10% cutoffs [9]. The role of PR-negative status in the context of RS and nodal staging remains unclear. We performed an observational cohort study to evaluate the association of PR status with RS and the magnitude of chemotherapy benefit on survival.

## Method

This study was approved under the protocol (BDR-131220) by Roswell Park Comprehensive Cancer Center, and follows the Strengthening the Reporting of Observational Studies in Epidemiology (STROBE) reporting guideline.

### Patient selection and variable definition

The National Cancer Database (NCDB) was queried for women diagnosed between 2010 and 2017 with ER-positive, HER2-negative, pT1-3N0-1aM0 breast cancer who underwent surgery and adjuvant endocrine therapy with available RS. If 1% or greater cells stained positively, they were considered PR-positive. Otherwise, they were considered PR-negative. Variables of interest were facility type, age at diagnosis, race, medical insurance, income and education level, Charlson-Deyo Comorbidity Score (CDS), year of diagnosis, histology, tumor grade, T and N staging, recurrence score, progesterone receptor status, lymphovascular space invasion (LVSI), surgery, surgical margin, radiation therapy, and chemotherapy. Age was stratified by above versus below 50 years of age. Education and income levels were determined based on the 2016 American Community Survey data between 2012 and 2016. Such levels were the percentages of adults who did not graduate from high school and the median household income adjusted for 2016 inflation, respectively, in each patient’s zip code in the United States. High versus low neighborhood-level income and education were determined by the median value of 10.9% and $50,353, respectively. All missing values were labeled as unknown. Other variables, such as performance status, type and duration of systemic therapy, toxicity profile, breast cancer-specific mortality, tumor recurrence, were not captured in the NCDB.

### Statistical analysis

Our primary endpoint was overall survival (OS), the time interval between diagnosis and the last follow up or death from any cause. Baseline characteristics were evaluated using Fisher exact test and Mann–Whitney U test as appropriate. Logistic multivariable analysis (MVA) was performed based on baseline patient and tumor characteristics to identify variables associated with PR-negative tumors. Kaplan–Meier method, log-rank test, and Cox MVA models including all clinically relevant variables were performed for OS. Crude odds ratio and hazards ratio results were reported using logistic and Cox univariable analysis (UVA). Variables included for logistic and Cox MVA are listed in eTable 1 and eTable 2, respectively. For patients diagnosed in 2017, OS were not captured in the NCDB, and these patients were excluded for OS analysis.

Interaction term analysis was performed to assess any heterogeneous association of PR status and chemotherapy receipt with OS. If the interaction term was significant, subgroup analyses were performed to compare the magnitude of the effect of chemotherapy and PR status. To reduce the selection bias and further evaluate the subgroup analysis results, propensity score matching was performed based on all variables of interest. Matching was performed using nearest neighbor method in a 1:1 ratio without replacements. The standardized differences of all variables were less than 0.1, indicating adequate match with negligible differences between arms [10]. To address immortal time bias, Cox MVA analyses were repeated after excluding patients with post-diagnosis OS of less than 6 months. Additional subgroup analysis was performed among those with RS ≤ 25 by repeating logistic and Cox MVA.

All p values were two-sided, and p values less than 0.05 were considered statistically significant. All analyses were performed using R (version 4.0.3, R Project for Statistical Computing, Vienna, Austria).

## Results

A total of 143,828 women (median [interquartile range (IQR)] age, 60 [51–67] years) met our criteria (Table 1). Of these, 130,349 (90.6%) and 13,479 (9.4%) patients had PR-positive and PR-negative tumors, respectively. Median (IQR) follow up was 51.5 months (34.8–71.9).

**Table 1:** Baseline characteristics

	PR +		PR-		
	N	%	N	%	*P*
PR					
Positive	123,751	100.0	0	0.0	
Negative	0	0.0	12,769	100.0	
Age					< 0.001
< 50 years	27,107	20.8	1155	8.6	
50 years or older	103,242	79.2	12,324	91.4	
Race					< 0.001
Non-Hispanic White	106,157	81.4	10,744	79.7	
Hispanic White	7437	5.7	734	5.4	
Black	9686	7.4	1352	10.0	
Asian/Pacific Islander	4946	3.8	447	3.3	
Other	1064	0.8	96	0.7	
Not available	1059	0.8	106	0.8	
Facility					< 0.001
Nonacademic	82,586	63.4	8612	63.9	
Academic	44,100	33.8	4643	34.4	
Not available	3663	2.8	224	1.7	
Insurance					< 0.001
None	1551	1.2	179	1.3	
Private	79,056	60.6	7181	53.3	
Government	48,434	37.2	5993	44.5	
Not available	1308	1.0	126	0.9	
Income					0.01
Above median	75,920	58.2	7802	57.9	
Below median	35,120	26.9	3767	27.9	
Not available	19,309	14.8	1910	14.2	
Education					< 0.001
Above median	70,707	54.2	7175	53.2	
Below median	40,507	31.1	4415	32.8	
Not available	19,135	14.7	1889	14.0	
CDS					< 0.001
0	110,215	84.6	11,207	83.1	
1	16,208	12.4	1824	13.5	
2 +	3926	3.0	448	3.3	
Year					0.001
Median	2014		2014		
IQR	2012–2016		2012–2016		
Histology					< 0.001
Ductal or lobular carcinoma	111,903	85.8	11,764	87.3	
Other	18,446	14.2	1715	12.7	
T staging					< 0.001
1	97,532	74.8	9631	71.5	
2	31,013	23.8	3621	26.9	
3	1804	1.4	227	1.7	
N staging					< 0.001
0	110,421	84.7	11,897	88.3	
1a	19,928	15.3	1582	11.7	
Grade					< 0.001
1	37,224	28.6	2871	21.3	
2	70,043	53.7	6533	48.5	
3	18,379	14.1	3563	26.4	
Other	58	0.0	21	0.2	
Not available	4645	3.6	491	3.6	
RS					< 0.001
0–15	68,683	52.7	1925	14.3	
16–25	46,723	35.8	5394	40.0	
> 25	14,943	11.5	6160	45.7	
LVSI					< 0.001
No	99,783	76.6	10,446	77.5	
Yes	16,081	12.3	1526	11.3	
Not available	14,485	11.1	1507	11.2	
Chemotherapy					< 0.001
No	105,902	81.2	7435	55.2	
Yes	24,447	18.8	6044	44.8	
Radiation					0.36
No	40,291	30.9	4243	31.5	
Yes	88,630	68.0	9094	67.5	
Not available	1428	1.1	142	1.1	
Surgery					0.56
Lumpectomy	88,283	67.7	9161	68.0	
Mastectomy	42,039	32.3	4314	32.0	
Other	27	0.0	4	0.0	
Margin					0.02
Negative	126,053	96.7	12,990	96.4	
Positive	3856	3.0	451	3.3	
Not available	440	0.3	38	0.3	

*PR* Progesterone receptor, *N* Number, *CDS* Charlson-Deyo comorbidity score, *IQR* Interquartile range, *RS* 21-gene recurrence score, *LVSI* Lymphovascular space invasion

### Logistic and Cox MVA

Logistic MVA showed that PR-negative status was associated with higher RS (> 25: aOR 16.15, 95% CI 15.23–17.13, *p* < 0.001; eTable 1 in the Supplement). Cox MVA showed that PR-negative status was associated with worse OS (adjusted hazards ratio [aHR] 1.20, 95% CI 1.10–1.31, *p* < 0.001; eTable 2 in the Supplement). Consistent crude results were observed using logistic (odds ratio [OR] 14.87, 95% CI 14.07–15.73, *p* < 0.001) and Cox UVA (hazards ratio [HR] 1.52, 95% CI 1.42–1.62, *p* < 0.001).

### Interaction term and subgroup analyses

**Fig. 1:**
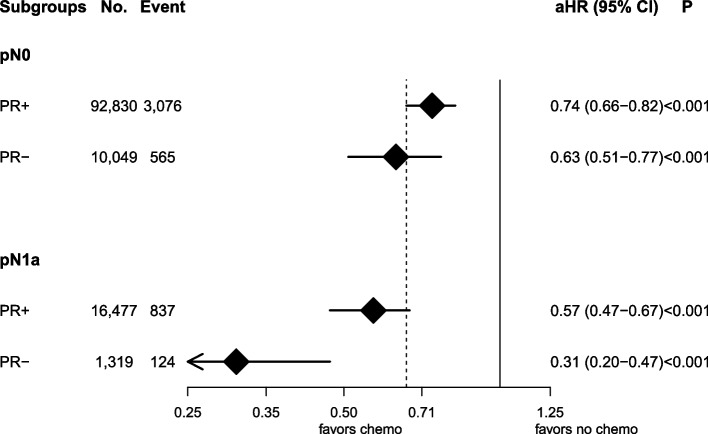
Forest plot of overall survival associated with chemotherapy stratified by progesterone receptor status and nodal staging using multivariable Cox regression. Dotted vertical line represents a hazards ratio of 0.66 associated with chemotherapy use for the entire cohort. No.: number of patients; aHR: adjusted hazards ratio; CI: confidence interval; PR: progesterone receptor; chemo: chemotherapy

There was no interaction of PR status with RS (*p* = 0.42), chemotherapy receipt (*p* = 0.30), or nodal staging (*p* = 0.13). While its three-way interaction with RS and chemotherapy was not statistically significant (*p* = 0.53), there was an interaction with nodal staging and chemotherapy (*p* = 0.049). Subgroup analyses using Cox MVA showed the magnitude of the benefit of chemotherapy was greater among those with PR-negative node-positive tumors than PR-positive node-positive tumors, while it was comparable among those with node-negative tumors regardless of PR status (Fig. 1).

### Propensity score matching analysis

**Fig. 2:**
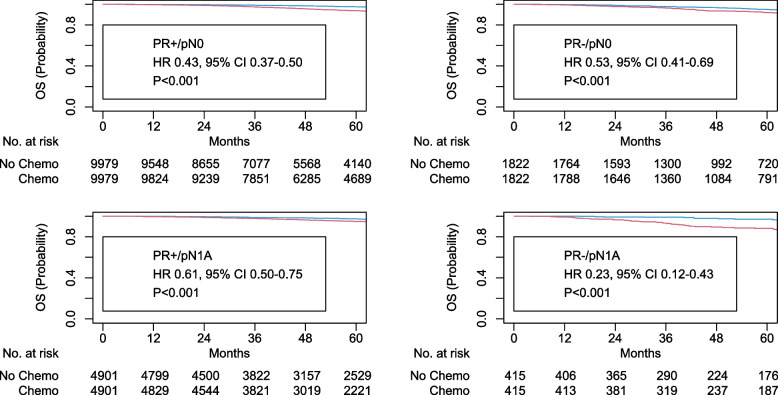
Kaplan Meier plots for overall survival associated with chemotherapy stratified by progesterone receptor status and nodal staging after propensity score matching, Red: no chemotherapy; blue: chemotherapy; PR: progesterone receptor; OS: overall survival; HR: hazards ratio; 95% CI: 95% confidence interval; chemo: chemotherapy

To further evaluate such subgroups, propensity score matching was performed between those with versus without chemotherapy stratified by PR status and nodal staging. Similar findings were observed after propensity score matching. The magnitude of chemotherapy benefits was greater for those with node-positive tumors (pN1a, PR-positive: HR 0.61, 95% CI 0.50–0.75, *p* < 0.001; pN1a, PR-negative: HR 0.23, 95% CI 0.12–0.43, *p* < 0.001; eTable 3 in the Supplement and Fig. 2) compared to those with node-negative tumors (pN0, PR-positive: HR 0.43, 95% CI 0.37–0.50, *p* < 0.001; pN0, PR-negative: HR 0.53, 95% CI 0.41–0.69, *p* < 0.001; eTable 4 in the Supplement and Fig. 2).

### Subgroup analysis to address immortal time bias

After excluding those with post-diagnosis OS of less than 6 months, PR-negative status remained associated with worse OS (aHR 1.20, 95% CI 1.10–1.31, *p* < 0.001). Interaction with chemotherapy and nodal staging remained to be statistically significant (*p* = 0.037) with similar findings on subgroup analyses (pN0, PR-positive: aHR 0.74, 95% CI 0.66–0.83, *p* < 0.001; pN0, PR-negative: aHR 0.64, 95% CI 0.52–0.79, *p* < 0.001; pN1a, PR-positive: aHR 0.57, 95% CI 0.48–0.68, *p* < 0.001; pN1a, PR-negative: aHR 0.31, 95% CI 0.20–0.47, *p* < 0.001).

### *Subgroup analysis among those with RS* ≤ *25*

On logistic MVA, PR-negative status remained statistically significant for its association with RS 16–25 compared to RS ≤ 15 (aOR 4.45, 95% CI 4.21–4.71, *p* < 0.001). On Cox MVA, however, PR-negative status was no longer associated with OS (aHR 1.07, 95% CI 0.97–1.19, *p* = 0.18), and interaction term analysis among PR status, chemotherapy use, and nodal staging was not statistically significant (interaction *p* = 0.64).

## Discussion

To our knowledge, this is the largest study based on nationwide oncology database to suggest that PR-negative status was independently correlated with higher RS and worse OS. It also suggested that, even after adjusting for age and RS, PR-negative tumors were associated with greater OS benefits from chemotherapy among patients with pN1a, but not pN0, breast cancer.

The proportion of PR-negative tumors in our study (9.4%) was comparable to the United States population-based study (9.1%) [11] as well as prospective trials including TAILORx (10.0%) and RxPONDER (5.7%) trials [7, 8]. Such observation suggests the overall consistency of PR status in patient population among the hospital registry-based database, the population-based database, and prospective trials. However, such proportions were lower than 23% of tumors being PR-negative reported by the study from the United Kingdom and Ireland. This inconsistency may be in part explained by allowing up to 10% of cells stained positively to be considered as PR-negative tumors [12], as opposed to less than 1% of cells as defined in our study.

Our findings on the PR-negative tumors associated with aggressive tumor biology as suggested by high RS and worse OS are consistent with other studies suggesting worse prognosis and survival outcomes [13–15]. Although 5-year survival rates for breast cancer have been improving in the United States, ER-positive breast cancers that are PR-negative still have a significantly lower OS compared to ER- and PR-positive tumors [14, 16, 17]. Patients with ER-positive breast cancers with low or no PR expression also have a greater risk of tumor recurrence [15, 17, 18]. Prior population study has shown that when ER-positive breast cancer recurs, 26% of the tumors will convert from PR-positive to PR-negative status, suggesting that a loss of PR expression is indicative of refractory response to hormone therapy responsiveness and further disease progression [14].

In our study, reasons for PR-negative tumors with greater OS benefits from chemotherapy among node-positive tumors compared to node-negative tumors remain unclear. At the absence of systemic therapy, node-positive tumors have been shown to be more aggressive with higher distant recurrence rates [19]. Although not statistically powered for subgroup analysis, KEYNOTE-522 trial also showed the treatment with pembrolizumab was associated with numerically higher survival outcomes among node-positive tumors compared to node-negative counterparts [20]. Patients with ER-positive, PR-negative tumors had comparable, poor outcomes similar to triple negative tumors [2], and given such tumor biology, treatment intensification with chemotherapy is associated with survival benefits for node-positive tumors.

Limitations of our study include inherent biases due to its retrospective nature. In addition, given the nature of OS as an endpoint, a short median follow-up of 51.5 months, and a lack of tumor recurrence data, the number of events was too low for subgroup analysis especially among patients younger than 50 years of age. Although most patients were non-Hispanic White in our cohort, heterogeneous characteristics seen between patients with PR-positive and PR-negative tumors (Table 1) may be in part due to sociodemographic factors and likely inter-related with one another. Additional analyses were not performed to investigate complex interactions among such variables, since they were beyond the scope of this study. In addition, as shown in our subgroup analysis among those with RS ≤ 25 suggesting a lack of association between PR status and OS, our study was likely underpowered to evaluate whether PR-negative status as an adverse prognostic factor would be valid among subgroups established based on menopausal status, age, and RS cutoffs by TAILORx and RxPONDER trials [7, 8].

## Conclusion

Our study suggests that PR-negative status is associated with high RS and worse OS. PR-negative tumors were shown to benefit more from chemotherapy than PR-positive tumors in the node-positive setting. Further investigations are warranted to tailor systemic therapies among PR-negative tumors.

## Supplementary Information

**Additional file 1: eTable 1.**Logistic multivariable analysis for progesterone receptor status. **eTable 2**. Cox multivariable analysis for overall survival. **eTable 3**. Baseline characteristics for node-positive tumors after propensity score matching. **eTable 4**. Baseline characteristics for node-negative tumors after propensity score matching. 

## Data Availability

The primary dataset (National Cancer Database) is available publicly for investigators associated with Commission on Cancer-accredited programs through the American College of Surgeons (https://www.facs.org/quality-programs/cancer/ncdb).
