# Supplementary material for: Association of progesterone receptor status with 21-gene recurrence score and survival among patients with estrogen receptor-positive breast cancer
Source: BMC Cancer. 2023 Apr 11;23:330. doi: 10.1186/s12885-023-10796-4 (PMC10088125; doi:10.1186/s12885-023-10796-4)
Supplement: Supplementary file 1 — Additional file 1: eTable 1.Logistic multivariable analysis for progesterone receptor status. eTable 2. Cox multivariable analysis for overall survival. eTable 3. Baseline characteristics for node-positive tumors after propensity score matching. eTable 4. Baseline characteristics for node-negative tumors after propensity score matching. [file 12885_2023_10796_MOESM1_ESM.docx]

**Supplementary Online Content**

**eTable 1**. Logistic multivariable analysis for progesterone receptor status

**eTable 2**. Cox multivariable analysis for overall survival

**eTable 3**. Baseline characteristics for node-positive tumors after propensity score matching

**eTable 4**. Baseline characteristics for node-negative tumors after propensity score matching

**eTable 1**. Logistic multivariable analysis for progesterone receptor status

|  | aOR | 95% CI | P |
| --- | --- | --- | --- |
| RS |  |  |  |
| 0-15 | Reference |  |  |
| 16-25 | 4.29 | 4.06-4.52 | <0.001 |
| >25 | 16.15 | 15.23-17.13 | <0.001 |
|  |  |  |  |
| Age |  |  |  |
| <50 years | Reference |  |  |
| 50 years or older | 2.95 | 2.75-3.17 | <0.001 |
|  |  |  |  |
| Race |  |  |  |
| Non-Hispanic White | Reference |  |  |
| Hispanic White | 1.02 | 0.94-1.11 | 0.69 |
| Black | 1.26 | 1.18-1.35 | <0.001 |
| Asian/Pacific Islander | 0.99 | 0.89-1.10 | 0.86 |
| Other | 1.07 | 0.85-1.33 | 0.55 |
|  |  |  |  |
| Facility |  |  |  |
| Nonacademic | Reference |  |  |
| Academic | 1.02 | 0.98-1.06 | 0.36 |
|  |  |  |  |
| Insurance |  |  |  |
| None | Reference |  |  |
| Private | 0.79 | 0.67-0.93 | 0.005 |
| Government | 0.94 | 0.80-1.12 | 0.51 |
|  |  |  |  |
| Income |  |  |  |
| Above median | Reference |  |  |
| Below median | 0.91 | 0.87-0.96 | <0.001 |
|  |  |  |  |
| Education |  |  |  |
| Above median | Reference |  |  |
| Below median | 1.02 | 0.97-1.07 | 0.41 |
|  |  |  |  |
| CDS |  |  |  |
| 0 | Reference |  |  |
| 1 | 0.98 | 0.92-1.03 | 0.42 |
| 2+ | 0.94 | 0.84-1.04 | 0.22 |
|  |  |  |  |
| Year |  |  |  |
| For every 1 year increase | 1 | 0.99-1.00 | 0.28 |
|  |  |  |  |
| Histology |  |  |  |
| Ductal or lobular carcinoma | Reference |  |  |
| Other | 1.03 | 0.97-1.08 | 0.39 |
|  |  |  |  |
| T staging |  |  |  |
| 1 | Reference |  |  |
| 2 | 1.02 | 0.98-1.07 | 0.31 |
| 3 | 1.48 | 1.27-1.72 | <0.001 |
|  |  |  |  |
| N staging |  |  |  |
| 0 | Reference |  |  |
| 1a | 0.79 | 0.74-0.84 | <0.001 |
|  |  |  |  |
| Grade |  |  |  |
| 1 | Reference |  |  |
| 2 | 0.89 | 0.85-0.94 | <0.001 |
| 3 | 0.83 | 0.78-0.89 | <0.001 |
| Other | 1.5 | 0.85-2.56 | 0.15 |
|  |  |  |  |
| LVSI |  |  |  |
| No | Reference |  |  |
| Yes | 0.79 | 0.75-0.85 | <0.001 |

aOR: adjusted odds ratio; CI: confidence interval; RS: 21-gene recurrence score; CDS: Charlson-Deyo score; LVSI: lymphovascular space invasion

**eTable 2**. Cox multivariable analysis for overall survival

|  | aHR | 95% CI | P |
| --- | --- | --- | --- |
| Tumor receptor | |  |  |
| PR+ | Reference |  |  |
| PR- | 1.2 | 1.10-1.31 | <0.001 |
|  |  |  |  |
| Age |  |  |  |
| <50 years | Reference |  |  |
| 50 years or older | 1.77 | 1.57-1.99 | <0.001 |
|  |  |  |  |
| Race |  |  |  |
| Non-Hispanic White | Reference |  |  |
| Hispanic White | 0.82 | 0.72-0.94 | 0.003 |
| Black | 1.06 | 0.96-1.17 | 0.28 |
| Asian/Pacific Islander | 0.62 | 0.50-0.77 | <0.001 |
| Other | 0.6 | 0.37-0.97 | 0.04 |
|  |  |  |  |
| Facility |  |  |  |
| Nonacademic | Reference |  |  |
| Academic | 0.75 | 0.71-0.81 | <0.001 |
|  |  |  |  |
| Insurance |  |  |  |
| None | Reference |  |  |
| Private | 0.61 | 0.47-0.79 | <0.001 |
| Government | 1.42 | 1.10-1.83 | 0.007 |
|  |  |  |  |
| Income |  |  |  |
| Above median | Reference |  |  |
| Below median | 1.15 | 1.06-1.24 | <0.001 |
|  |  |  |  |
| Education |  |  |  |
| Above median | Reference |  |  |
| Below median | 1.05 | 0.98-1.13 | 0.16 |
|  |  |  |  |
| CDS |  |  |  |
| 0 | Reference |  |  |
| 1 | 1.64 | 1.52-1.76 | <0.001 |
| 2+ | 2.64 | 2.37-2.94 | <0.001 |
|  |  |  |  |
| Year |  |  |  |
| For every 1 year increase | 1.07 | 1.05-1.09 | <0.001 |
|  |  |  |  |
| Histology |  |  |  |
| Ductal or lobular carcinoma | Reference |  |  |
| Other | 0.96 | 0.88-1.04 | 0.32 |
|  |  |  |  |
| T staging |  |  |  |
| 1 | Reference |  |  |
| 2 | 1.5 | 1.41-1.60 | <0.001 |
| 3 | 2.14 | 1.75-2.62 | <0.001 |
|  |  |  |  |
| N staging |  |  |  |
| 0 | Reference |  |  |
| 1a | 1.57 | 1.45-1.70 | <0.001 |
|  |  |  |  |
| Grade |  |  |  |
| 1 | Reference |  |  |
| 2 | 1.13 | 1.04-1.22 | 0.002 |
| 3 | 1.51 | 1.37-1.66 | <0.001 |
| Other | 0.97 | 0.40-2.33 | 0.94 |
|  |  |  |  |
| RS |  |  |  |
| 0-15 | Reference |  |  |
| 16-25 | 1.18 | 1.10-1.26 | <0.001 |
| >25 | 2.21 | 2.01-2.43 | <0.001 |
|  |  |  |  |
| LVSI |  |  |  |
| No | Reference |  |  |
| Yes | 1.2 | 1.11-1.31 | <0.001 |
|  |  |  |  |
| Chemotherapy | |  |  |
| No | Reference |  |  |
| Yes | 0.66 | 0.61-0.72 | <0.001 |
|  |  |  |  |
| Radiation |  |  |  |
| No | Reference |  |  |
| Yes | 0.59 | 0.54-0.66 | <0.001 |
|  |  |  |  |
| Surgery |  |  |  |
| Lumpectomy | Reference |  |  |
| Mastectomy | 0.75 | 0.68-0.84 | <0.001 |
| Other | 1.8 | 0.45-7.21 | 0.41 |
|  |  |  |  |
| Margin |  |  |  |
| Negative | Reference |  |  |
| Positive | 1.13 | 0.96-1.33 | 0.13 |

aHR: adjusted hazards ratio; CI: confidence interval; PR: progesterone receptor; RS: 21-gene recurrence score; CDS: Charlson-Deyo score; LVSI: lymphovascular space invasion

**eTable 3**. Baseline characteristics for node-positive tumors after propensity score matching

|  | pN1a, PR+ | |  |  |  | pN1a, PR- | |  |  |  |
| --- | --- | --- | --- | --- | --- | --- | --- | --- | --- | --- |
|  | No Chemo | | Chemo |  |  | No Chemo | | Chemo |  |  |
|  | N | % | N | % | Std Diff | N | % | N | % | Std Diff |
| Chemo |  |  |  |  |  |  |  |  |  |  |
| No | 4901 | 100.0 | 0 | 0.0 |  | 415 | 100.0 | 0 | 0.0 |  |
| Yes | 0 | 0.0 | 4901 | 100.0 |  | 0 | 0.0 | 415 | 100.0 |  |
|  |  |  |  |  |  |  |  |  |  |  |
| Age |  |  |  |  | 0.009 |  |  |  |  | 0.04 |
| <50 years | 1180 | 24.1 | 1198 | 24.4 |  | 20 | 4.8 | 24 | 5.8 |  |
| 50 years or older | 3721 | 75.9 | 3703 | 75.6 |  | 395 | 95.2 | 391 | 94.2 |  |
|  |  |  |  |  |  |  |  |  |  |  |
| Race |  |  |  |  | 0.009 |  |  |  |  | 0.03 |
| Non-Hispanic White | 3987 | 81.4 | 3940 | 80.4 |  | 335 | 80.7 | 330 | 79.5 |  |
| Hispanic White | 302 | 6.2 | 303 | 6.2 |  | 20 | 4.8 | 21 | 5.1 |  |
| Black | 363 | 7.4 | 381 | 7.8 |  | 42 | 10.1 | 43 | 10.4 |  |
| Asian/Pacific Islander | 165 | 3.4 | 198 | 4.0 |  | 11 | 2.7 | 13 | 3.1 |  |
| Other | 48 | 1.0 | 48 | 1.0 |  | 4 | 1.0 | 4 | 1.0 |  |
| Not available | 36 | 0.7 | 31 | 0.6 |  | 3 | 0.7 | 4 | 1.0 |  |
|  |  |  |  |  |  |  |  |  |  |  |
| Facility |  |  |  |  | 0.02 |  |  |  |  | 0.03 |
| Nonacademic | 3025 | 61.7 | 2962 | 60.4 |  | 269 | 64.8 | 264 | 63.6 |  |
| Academic | 1741 | 35.5 | 1791 | 36.5 |  | 144 | 34.7 | 148 | 35.7 |  |
| Not available | 135 | 2.8 | 148 | 3.0 |  | 2 | 0.5 | 3 | 0.7 |  |
|  |  |  |  |  |  |  |  |  |  |  |
| Insurance |  |  |  |  | 0.01 |  |  |  |  | 0.02 |
| None | 62 | 1.3 | 67 | 1.4 |  | 3 | 0.7 | 6 | 1.4 |  |
| Private | 3281 | 66.9 | 3244 | 66.2 |  | 205 | 49.4 | 203 | 48.9 |  |
| Government | 1523 | 31.1 | 1548 | 31.6 |  | 205 | 49.4 | 204 | 49.2 |  |
| Not available | 35 | 0.7 | 42 | 0.9 |  | 2 | 0.5 | 2 | 0.5 |  |
|  |  |  |  |  |  |  |  |  |  |  |
| Income |  |  |  |  | 0.003 |  |  |  |  | 0.02 |
| Above median | 2899 | 59.2 | 2924 | 59.7 |  | 247 | 59.5 | 241 | 58.1 |  |
| Below median | 1293 | 26.4 | 1273 | 26.0 |  | 117 | 28.2 | 120 | 28.9 |  |
| Not available | 709 | 14.5 | 704 | 14.4 |  | 51 | 12.3 | 54 | 13.0 |  |
|  |  |  |  |  |  |  |  |  |  |  |
| Education |  |  |  |  | 0.003 |  |  |  |  | 0.02 |
| Above median | 2680 | 54.7 | 2694 | 55.0 |  | 221 | 53.3 | 210 | 50.6 |  |
| Below median | 1518 | 31.0 | 1509 | 30.8 |  | 143 | 34.5 | 151 | 36.4 |  |
| Not available | 703 | 14.3 | 698 | 14.2 |  | 51 | 12.3 | 54 | 13.0 |  |
|  |  |  |  |  |  |  |  |  |  |  |
| CDS |  |  |  |  | 0.03 |  |  |  |  | 0.02 |
| 0 | 4226 | 86.2 | 4167 | 85.0 |  | 332 | 80.0 | 332 | 80.0 |  |
| 1 | 542 | 11.1 | 588 | 12.0 |  | 69 | 16.6 | 65 | 15.7 |  |
| 2+ | 133 | 2.7 | 146 | 3.0 |  | 14 | 3.4 | 18 | 4.3 |  |
|  |  |  |  |  |  |  |  |  |  |  |
| Year |  |  |  |  | 0.01 |  |  |  |  | 0.002 |
| 2006-2013 | 1966 | 40.1 | 1936 | 39.5 |  | 144 | 34.7 | 149 | 35.9 |  |
| 2014-2016 | 2920 | 59.6 | 2953 | 60.3 |  | 270 | 65.1 | 265 | 63.9 |  |
| Not available | 15 | 0.3 | 12 | 0.2 |  | 1 | 0.2 | 1 | 0.2 |  |
|  |  |  |  |  |  |  |  |  |  |  |
| Histology |  |  |  |  | 0.001 |  |  |  |  | 0.01 |
| Ductal or lobular carcinoma | 4259 | 86.9 | 4258 | 86.9 |  | 353 | 85.1 | 351 | 84.6 |  |
| Other | 642 | 13.1 | 643 | 13.1 |  | 62 | 14.9 | 64 | 15.4 |  |
|  |  |  |  |  |  |  |  |  |  |  |
| T staging |  |  |  |  | 0.02 |  |  |  |  | 0.01 |
| 1 | 2858 | 58.3 | 2800 | 57.1 |  | 241 | 58.1 | 242 | 58.3 |  |
| 2 | 1862 | 38.0 | 1920 | 39.2 |  | 153 | 36.9 | 154 | 37.1 |  |
| 3 | 181 | 3.7 | 181 | 3.7 |  | 21 | 5.1 | 19 | 4.6 |  |
|  |  |  |  |  |  |  |  |  |  |  |
| LVSI |  |  |  |  | 0.01 |  |  |  |  | 0.01 |
| No | 2682 | 54.7 | 2583 | 52.7 |  | 217 | 52.3 | 220 | 53.0 |  |
| Yes | 1637 | 33.4 | 1720 | 35.1 |  | 140 | 33.7 | 139 | 33.5 |  |
| Not available | 582 | 11.9 | 598 | 12.2 |  | 58 | 14.0 | 56 | 13.5 |  |
|  |  |  |  |  |  |  |  |  |  |  |
| Grade |  |  |  |  | 0.02 |  |  |  |  | 0.02 |
| 1 | 1034 | 21.1 | 1014 | 20.7 |  | 96 | 23.1 | 89 | 21.4 |  |
| 2 | 2970 | 60.6 | 2937 | 59.9 |  | 238 | 57.3 | 241 | 58.1 |  |
| 3 | 735 | 15.0 | 778 | 15.9 |  | 64 | 15.4 | 68 | 16.4 |  |
| Other | 1 | 0.0 | 1 | 0.0 |  | 1 | 0.2 | 1 | 0.2 |  |
| Not available | 161 | 3.3 | 171 | 3.5 |  | 16 | 3.9 | 16 | 3.9 |  |
|  |  |  |  |  |  |  |  |  |  |  |
| RS |  |  |  |  | 0.01 |  |  |  |  | 0.009 |
| 0-15 | 1358 | 27.7 | 1447 | 29.5 |  | 19 | 4.6 | 21 | 5.1 |  |
| 16-25 | 3083 | 62.9 | 2938 | 59.9 |  | 251 | 60.5 | 249 | 60.0 |  |
| >25 | 460 | 9.4 | 516 | 10.5 |  | 145 | 34.9 | 145 | 34.9 |  |
|  |  |  |  |  |  |  |  |  |  |  |
| Radiation |  |  |  |  | 0.01 |  |  |  |  | 0.06 |
| No | 1147 | 23.4 | 1162 | 23.7 |  | 111 | 26.7 | 96 | 23.1 |  |
| Yes | 3677 | 75.0 | 3654 | 74.6 |  | 299 | 72.0 | 311 | 74.9 |  |
| Not available | 77 | 1.6 | 85 | 1.7 |  | 5 | 1.2 | 8 | 1.9 |  |
|  |  |  |  |  |  |  |  |  |  |  |
| Surgery |  |  |  |  | 0.007 |  |  |  |  | 0.09 |
| Lumpectomy | 2705 | 55.2 | 2688 | 54.8 |  | 243 | 58.6 | 262 | 63.1 |  |
| Mastectomy | 2196 | 44.8 | 2212 | 45.1 |  | 172 | 41.4 | 153 | 36.9 |  |
| Other | 0 | 0.0 | 1 | 0.0 |  | 0 | 0.0 | 0 | 0.0 |  |
|  |  |  |  |  |  |  |  |  |  |  |
| Margin |  |  |  |  | 0.007 |  |  |  |  | 0.07 |
| Negative | 4697 | 95.8 | 4673 | 95.3 |  | 396 | 95.4 | 393 | 94.7 |  |
| Positive | 187 | 3.8 | 209 | 4.3 |  | 19 | 4.6 | 21 | 5.1 |  |
| Not available | 17 | 0.3 | 19 | 0.4 |  | 0 | 0.0 | 1 | 0.2 |  |

PR: progesterone receptor; N: number; CDS: Charlson-Deyo comorbidity score; RS: 21-gene recurrence score; LVSI: lymphovascular space invasion; chemo: chemotherapy

**eTable 4**. Baseline characteristics for node-negative tumors after propensity score matching

|  | pN0, PR+ | |  |  |  | pN0, PR- | |  |  |  |
| --- | --- | --- | --- | --- | --- | --- | --- | --- | --- | --- |
|  | No Chemo | | Chemo | |  | No Chemo | | Chemo | |  |
|  | N | % | N | % | Std Diff | N | % | N | % | Std Diff |
| Chemo |  |  |  |  |  |  |  |  |  |  |
| No | 9979 | 100.0 | 0 | 0.0 |  | 1822 | 100.0 | 0 | 0.0 |  |
| Yes | 0 | 0.0 | 9979 | 100.0 |  | 0 | 0.0 | 1822 | 100.0 |  |
|  |  |  |  |  |  |  |  |  |  |  |
| Age |  |  |  |  | 0.009 |  |  |  |  | 0.01 |
| <50 years | 3014 | 30.2 | 3056 | 30.6 |  | 154 | 8.5 | 159 | 8.7 |  |
| 50 years or older | 6965 | 69.8 | 6923 | 69.4 |  | 1668 | 91.5 | 1663 | 91.3 |  |
|  |  |  |  |  |  |  |  |  |  |  |
| Race |  |  |  |  | 0.01 |  |  |  |  | 0.02 |
| Non-Hispanic White | 8091 | 81.1 | 7981 | 80.0 |  | 1456 | 79.9 | 1436 | 78.8 |  |
| Hispanic White | 579 | 5.8 | 605 | 6.1 |  | 105 | 5.8 | 105 | 5.8 |  |
| Black | 792 | 7.9 | 823 | 8.2 |  | 166 | 9.1 | 183 | 10.0 |  |
| Asian/Pacific Islander | 360 | 3.6 | 399 | 4.0 |  | 72 | 4.0 | 70 | 3.8 |  |
| Other | 78 | 0.8 | 84 | 0.8 |  | 11 | 0.6 | 14 | 0.8 |  |
| Not available | 79 | 0.8 | 87 | 0.9 |  | 12 | 0.7 | 14 | 0.8 |  |
|  |  |  |  |  |  |  |  |  |  |  |
| Facility |  |  |  |  | 0.006 |  |  |  |  | 0.03 |
| Nonacademic | 6180 | 61.9 | 6065 | 60.8 |  | 1166 | 64.0 | 1144 | 62.8 |  |
| Academic | 3278 | 32.8 | 3380 | 33.9 |  | 633 | 34.7 | 648 | 35.6 |  |
| Not available | 521 | 5.2 | 534 | 5.4 |  | 23 | 1.3 | 30 | 1.6 |  |
|  |  |  |  |  |  |  |  |  |  |  |
| Insurance |  |  |  |  | 0.003 |  |  |  |  | 0.04 |
| None | 131 | 1.3 | 152 | 1.5 |  | 26 | 1.4 | 28 | 1.5 |  |
| Private | 6888 | 69.0 | 6840 | 68.5 |  | 926 | 50.8 | 960 | 52.7 |  |
| Government | 2860 | 28.7 | 2877 | 28.8 |  | 847 | 46.5 | 812 | 44.6 |  |
| Not available | 100 | 1.0 | 110 | 1.1 |  | 23 | 1.3 | 22 | 1.2 |  |
|  |  |  |  |  |  |  |  |  |  |  |
| Income |  |  |  |  | 0.005 |  |  |  |  | 0.03 |
| Above median | 5820 | 58.3 | 5870 | 58.8 |  | 1054 | 57.8 | 1039 | 57.0 |  |
| Below median | 2754 | 27.6 | 2722 | 27.3 |  | 523 | 28.7 | 517 | 28.4 |  |
| Not available | 1405 | 14.1 | 1387 | 13.9 |  | 245 | 13.4 | 266 | 14.6 |  |
|  |  |  |  |  |  |  |  |  |  |  |
| Education |  |  |  |  | 0.004 |  |  |  |  | 0.04 |
| Above median | 5495 | 55.1 | 5493 | 55.0 |  | 978 | 53.7 | 964 | 52.9 |  |
| Below median | 3094 | 31.0 | 3111 | 31.2 |  | 602 | 33.0 | 594 | 32.6 |  |
| Not available | 1390 | 13.9 | 1375 | 13.8 |  | 242 | 13.3 | 264 | 14.5 |  |
|  |  |  |  |  |  |  |  |  |  |  |
| CDS |  |  |  |  | 0.006 |  |  |  |  | 0.01 |
| 0 | 8612 | 86.3 | 8595 | 86.1 |  | 1522 | 83.5 | 1514 | 83.1 |  |
| 1 | 1161 | 11.6 | 1168 | 11.7 |  | 237 | 13.0 | 244 | 13.4 |  |
| 2+ | 206 | 2.1 | 216 | 2.2 |  | 63 | 3.5 | 64 | 3.5 |  |
|  |  |  |  |  |  |  |  |  |  |  |
| Year |  |  |  |  | 0.01 |  |  |  |  | <0.001 |
| 2006-2013 | 5654 | 56.7 | 5554 | 55.7 |  | 959 | 52.6 | 963 | 52.9 |  |
| 2014-2016 | 4219 | 42.3 | 4310 | 43.2 |  | 848 | 46.5 | 844 | 46.3 |  |
| Not available | 106 | 1.1 | 115 | 1.2 |  | 15 | 0.8 | 15 | 0.8 |  |
|  |  |  |  |  |  |  |  |  |  |  |
| Histology |  |  |  |  | 0.02 |  |  |  |  | 0.008 |
| Ductal or lobular carcinoma | 8849 | 88.7 | 8783 | 88.0 |  | 1593 | 87.4 | 1598 | 87.7 |  |
| Other | 1130 | 11.3 | 1196 | 12.0 |  | 229 | 12.6 | 224 | 12.3 |  |
|  |  |  |  |  |  |  |  |  |  |  |
| T staging |  |  |  |  | 0.001 |  |  |  |  | 0.02 |
| 1 | 7104 | 71.2 | 7101 | 71.2 |  | 1326 | 72.8 | 1311 | 72.0 |  |
| 2 | 2727 | 27.3 | 2726 | 27.3 |  | 466 | 25.6 | 481 | 26.4 |  |
| 3 | 148 | 1.5 | 152 | 1.5 |  | 30 | 1.6 | 30 | 1.6 |  |
|  |  |  |  |  |  |  |  |  |  |  |
| LVSI |  |  |  |  | 0.03 |  |  |  |  | <0.001 |
| No | 7671 | 76.9 | 7555 | 75.7 |  | 1456 | 79.9 | 1432 | 78.6 |  |
| Yes | 1251 | 12.5 | 1285 | 12.9 |  | 168 | 9.2 | 192 | 10.5 |  |
| Not available | 1057 | 10.6 | 1139 | 11.4 |  | 198 | 10.9 | 198 | 10.9 |  |
|  |  |  |  |  |  |  |  |  |  |  |
| Grade |  |  |  |  | 0.006 |  |  |  |  | 0.04 |
| 1 | 1682 | 16.9 | 1656 | 16.6 |  | 322 | 17.7 | 308 | 16.9 |  |
| 2 | 5295 | 53.1 | 5347 | 53.6 |  | 975 | 53.5 | 931 | 51.1 |  |
| 3 | 2575 | 25.8 | 2528 | 25.3 |  | 445 | 24.4 | 495 | 27.2 |  |
| Other | 6 | 0.1 | 12 | 0.1 |  | 3 | 0.2 | 3 | 0.2 |  |
| Not available | 421 | 4.2 | 436 | 4.4 |  | 77 | 4.2 | 85 | 4.7 |  |
|  |  |  |  |  |  |  |  |  |  |  |
| RS |  |  |  |  | 0.001 |  |  |  |  | 0.005 |
| 0-15 | 1009 | 10.1 | 1050 | 10.5 |  | 46 | 2.5 | 50 | 2.7 |  |
| 16-25 | 6283 | 63.0 | 6193 | 62.1 |  | 766 | 42.0 | 763 | 41.9 |  |
| >25 | 2687 | 26.9 | 2736 | 27.4 |  | 1010 | 55.4 | 1009 | 55.4 |  |
|  |  |  |  |  |  |  |  |  |  |  |
| Radiation |  |  |  |  | 0.01 |  |  |  |  | 0.01 |
| No | 3313 | 33.2 | 3261 | 32.7 |  | 633 | 34.7 | 614 | 33.7 |  |
| Yes | 6576 | 65.9 | 6616 | 66.3 |  | 1169 | 64.2 | 1186 | 65.1 |  |
| Not available | 90 | 0.9 | 102 | 1.0 |  | 20 | 1.1 | 22 | 1.2 |  |
|  |  |  |  |  |  |  |  |  |  |  |
| Surgery |  |  |  |  | 0.01 |  |  |  |  | 0.01 |
| Lumpectomy | 6571 | 65.8 | 6627 | 66.4 |  | 1218 | 66.8 | 1228 | 67.4 |  |
| Mastectomy | 3405 | 34.1 | 3350 | 33.6 |  | 604 | 33.2 | 594 | 32.6 |  |
| Other | 3 | 0.0 | 2 | 0.0 |  | 0 | 0.0 | 0 | 0.0 |  |
|  |  |  |  |  |  |  |  |  |  |  |
| Margin |  |  |  |  | 0.01 |  |  |  |  | 0.01 |
| Negative | 9624 | 96.4 | 9614 | 96.3 |  | 1755 | 96.3 | 1747 | 95.9 |  |
| Positive | 318 | 3.2 | 322 | 3.2 |  | 62 | 3.4 | 69 | 3.8 |  |
| Not available | 37 | 0.4 | 43 | 0.4 |  | 5 | 0.3 | 6 | 0.3 |  |

PR: progesterone receptor; N: number; CDS: Charlson-Deyo comorbidity score; RS: 21-gene recurrence score; LVSI: lymphovascular space invasion; chemo: chemotherapy
